# Safety and dosing of testosterone for hormone restoration in neutered dogs

**DOI:** 10.1186/s12917-025-04869-8

**Published:** 2025-07-09

**Authors:** Linda Brent, Florian Roeber, Eric Weber, Michael Chambers, Terry M. Nett, Elaine A. Lissner

**Affiliations:** 1 Parsemus Foundation, San Francisco, CA 94114 USA; 2 Invetus Pty Ltd, 95 Clarkes Road, Armidale, NSW 2350 Australia; 3 Animal Reproduction & Biotechnology Laboratory, Colorado State University, Fort Collins, CO 80523 USA

**Keywords:** Testosterone, Canine, Gonadectomy, Neuter, Spay-neuter syndrome, Hormone therapy

## Abstract

**Background:**

A lack of safety and dosing information on restoring hormone levels in neutered dogs has hindered treatment of spay-neuter syndrome, the diverse set of health and behavior conditions resulting from the loss of gonadal hormones. The purpose of this study was to provide basic information on the use of testosterone treatment in male neutered dogs using a target animal safety study design. Twelve previously neutered dogs were divided into four equal groups, receiving 0 (controls), 1x, 3x, or 5x the standard weekly dose of injectable testosterone cypionate (0.5 mg/kg) for 90 days. Bloodwork, including endocrine assays, body condition, prostate health, and qualitative behavioral observations were used to evaluate any changes in health during treatment.

**Results:**

Testosterone levels increased in a dose-dependent fashion, and luteinizing hormone decreased after a month of treatment in dogs receiving 5x the standard dose. Behavioral measures, Zambelli prostate health score, body condition scores, clinical evaluations, and routine blood hematology and chemistries showed minor variation over time or across groups. Two seizures were documented in a dog from group 3x with previous suspected idiopathic epilepsy.

**Conclusions:**

The results of the initial safety study indicated that weekly injectable testosterone therapy over a 3-month period was safe and capable of increasing testosterone levels in neutered dogs to within the normal range for intact dogs. The limitations of the study included small group sizes and no long-term follow up. A summary of the risks and recommendations for the use of testosterone restoration in dogs was provided.

**Supplementary Information:**

The online version contains supplementary material available at 10.1186/s12917-025-04869-8.

## Introduction

In many countries, surgical gonadectomy (removal of the ovaries in females and testicles in males) is common and considered part of responsible pet ownership. This form of sterilization removes the organs that produce reproductive hormones, including estradiol and testosterone, which initiates changes in the body beyond the inability to produce offspring.

As research has advanced, a better understanding of the risks and benefits of canine gonadectomy on lifelong physical and behavioral health is developing. In addition to eliminating risks associated with reproduction, the key benefits of removing the reproductive organs are eliminating or reducing the risk of diseases of those reproductive tissues (e.g., ovarian or testicular tumors). Gonadectomy also reduces the incidence of some diseases in males influenced by the hormones the organs normally produce, such as benign prostatic hyperplasia, yet may increase the risk of prostate carcinoma [1, 2]. Behaviors tightly linked to reproductive hormones, including pseudopregnancy and maternal aggression in females, and urine marking, roaming, and libido in males, are often reduced after gonadectomy [3].

The health risks of spaying and neutering include a higher prevalence of cancer, urinary incontinence, obesity, hypothyroidism, hypoadrenocorticism, and orthopedic diseases. Behavior changes such as increased aggression, fearfulness, anxiety, and reactivity have been reported [3]. These diverse conditions are herein collectively called “spay-neuter syndrome.” Risks may vary by breed, size, age, housing, care, and age at gonadectomy. The loss of reproductive hormones resulting from gonadectomy disrupts the negative feedback from gonadal hormones to the hypothalamus and pituitary, leading to increased levels of luteinizing hormone (LH) and follicle-stimulating hormone (FSH). High levels of LH can influence organ function and the development of cancer throughout the body [4].

Given the increasing knowledge about health risks of gonadectomy, alternative methods of sterilization that preserve hormones are becoming more common. Surgical options include hysterectomy and vasectomy [3, 5]. Nonsurgical options, including intraepididymal chemical castration [6] and testicular ultrasound in male dogs [7], have been studied. Dogs with longer gonadal hormone exposure, whether intact, sterilized with a gonad-sparing method, or spayed/neutered later in life, have fewer general health problems, fewer problematic and nuisance behaviors, and longer lifespans [8].

Unfortunately, little information exists for treatment of spay-neuter syndrome in dogs that have already undergone gonadectomy. Hormone replacement or restoration for dogs has received little research attention except for the treatment of urethral sphincter mechanism incontinence. Incontinence in female dogs is common after spaying [9] and has been treated successfully with conjugated estrogens, diethylstilbesterol, estriol, gonadotropin-releasing hormone (GnRH) agonists (e.g., deslorelin), or GnRH immunization [10–13], which reduces the high luteinizing hormone levels. Amitriptyline also proved to be an effective treatment for incontinence in spayed female dogs [14]. Urinary incontinence is less common in males after castration, with mixed success in treating the condition with testosterone [15, 16]. Data on hormone restoration to treat more diverse symptoms following castration involve only one case study describing the resolution of behavioral and orthopedic symptoms in a neutered mixed-breed dog [17].

For veterinarians to provide hormone therapy for the treatment of spay-neuter syndrome, basic information on the safety and dosing of medication is critical. The present study aimed to examine the safety of testosterone cypionate treatment in male neutered dogs to standardize the use of this drug for the management of spay-neuter syndrome. This exploratory study was conducted as a target animal safety study including controls and three treatment levels. The objectives were to determine any differences in health and behavior between groups or over time and if testosterone supplementation was related to any alterations in endocrine measures.

## Materials and methods

### Animals, study site, and housing

The subjects of the study included 6 neutered male beagles (mean weight = 18.2 kg; range 16.1–21.2 kg) and 6 foxhounds (mean weight 35.9 kg; range 30.7–42.9 kg). None of the dogs had been used in breeding previously. The average age of the dogs was 2.9 ± 1.8 years upon enrollment in the study. They were castrated at 7.7 ± 5.6 months of age and the average time between castration and study initiation was 2.2 ± 1.4 years.

The study was conducted at Invetus Wongabura Research Center in New South Wales, Australia. The research center owned the dogs and they were familiar with the staff, housing, and most of the other dogs at the facility. During the study, the dogs were socially housed in groups of three in their normal pens measuring a total of 1.5 m x 6 m (with equal areas inside and outside). The interior pens maintained the temperature between 16 and 29 °C and provided 8–15 volumetric changes of air per hour. Each pen was provided with a raised resting platform and an automatic water trough. The exterior pens were roofed, and dogs had free access to the interior and exterior pens. The dogs were exercised in socially compatible groups in outdoor grassed runs of approximately 500 m². The exercise period lasted for approximately 30 min per day when consistent with weather and protocol requirements.

Dogs were fed a commercially available dog chow (Cobber Working Dog, Victoria AU, with 26% protein, 18% fat) and water was available ad libitum. Routine health monitoring by trained staff included twice daily health observations and follow-up observation and treatment of any abnormal findings. All dogs received routine vaccinations and anthelmintics as needed. The beagle and foxhound breeds used in the study have pendulous ears which predisposed them to develop otitis externa, and treatment during the study was not considered an adverse event. All dogs were returned to the colony following the study.

### Study design

The overall design of the study was a negatively controlled target animal safety study. Fifteen dogs underwent a pre-enrollment veterinary examination 8 days before treatment (Day - 8), and 12 clinically healthy dogs were selected for the study based on favorable hematological and biochemical parameters, suitable demeanor, and veterinary clinical examination. Dogs were ranked based on bodyweight and breed, blocked into groups of four, and were then randomly assigned to one of the four treatment groups.

From Day - 8 to -1, twice daily (a.m./p.m.) observations of each individual dog’s behavior were conducted during routine husbandry activities. The qualitative observations established a baseline that served as a reference for future determination of behavioral change and any clinical signs of benign prostatic hyperplasia as referenced by the Zambelli Index Score [18] (see Table 1).

**Table 1:** Behavior observations recorded

Category	Reference behaviors recorded ^1^
Aggressive	Barking, Growling, Snarling, Biting towards other dogs or handlers
Fear	Shaking, Shivering, Howling, Freezing, Urinating
Excitability	Hyper-excitement, Jumping up in pen, Excessive vocalization
Other	Coprophagy, Tail-chasing, Excessive licking, Self-mutilation, Mounting, Elevated reproductive/dominance behavior
Feed consumption	Depressed, Anorexia, Weight loss
Defecation	Straining, Painful defecation, Inability to pass stool, Diarrhea
Urination	Urinary incontinence, Dysuria, Hematuria, Urethral leakage

^1^ Behavioral observations were conducted twice per day and scored as 1 = normal, 2 = moderately elevated or depressed, or 3 = highly abnormal

The various measures conducted during the study were scheduled to reduce the handling of the subjects. On all study days (0–90), each dog’s behavior was assessed twice daily as above. The observations included a qualitative assessment (normal, moderately elevated/depressed, or highly abnormal) for each category, specific notes about individuals involved in social interactions, and any non-normal events. The kennel staff performing the daily behavioral assessments were blinded to treatment groups.

Each week during the 3-month study, dogs were weighed, and clinical examinations were conducted. At day 0 and day 90, body condition scores according to the World Small Animal Veterinary Association [19] were determined, waist circumference was measured, and the Zambelli Symptom Index was assessed. Additional prostate evaluation (palpation, ultrasound) was not performed in this minimally invasive study design. Blood was collected via venipuncture prior to treatment on Days 0 (baseline), 7, 14, 28, 56, and Day 90 for hematology, biochemistry, and endocrinology. Thus, after baseline, evaluation of blood parameters was completed 1 week after each dose of testosterone. See Table 2 for the timeline of health and behavior measures collected and Table 3 for clinical examination and blood measures.

**Table 2:** Experimental design and data collection

Day	Weight	Clinical exam^1^	Blood collection^2^	Body condition^3^	Zambelli Symptom Index	Treatment
0	X	X	X (pre-treatment)	X	X	X
7	X	X	X			X
14	X	X	X			X
21	X	X				X
28	X	X	X			X
35	X	X				X
42	X	X				X
49	X	X				X
56	X	X	X			X
63	X	X				X
70	X	X				X
77	X	X				X
84	X	X				X
90	X	X	X	X	X	

^1^ Temperature, heart rate, respiration, injection site assessments. ^2^ CBC, biochemistry, endocrinology. ^3^ Body condition score, waist circumference

**Table 3:** Clinical examination, blood, and hormone measurements

Type	Measurement
Clinical Examination	Heart rate, Respiration rate, Temperature (rectal), Body weight, Body condition score, Waist circumference, Zambelli Symptom Index
Hematology/Biochemistry	White blood count (WBC), Hemoglobin (Hb), Red blood cell count (RBC), Hematocrit, Alkaline Phosphatase (ALP), Alanine aminotransferase (ALT), Urea nitrogen (BUN), Creatinine, Protein, Sodium, Potassium, Glucose
Endocrinology	Estradiol, Cortisol, Luteinizing hormone (LH), Progesterone, Testosterone, Thyroid stimulating hormone (TSH), Thyroid hormone (T4)

Following the weekly clinical exam, dogs were treated with a subcutaneous injection of a liquid formulation of 100 mg/mL testosterone cypionate (DEPO^®^-Testosterone, Pfizer) at 0x (Control), 1x (Group 1x), 3x (Group 3x), and 5x (Group 5x) the target dose of 0.5 mg/kg based on the dog’s weight prior to treatment.

### Blood and hormone analysis

Hematology and biochemistry assays were completed at Vetnostics (New South Wales, Australia) using standard procedures. Endocrine measures were completed at the Colorado State University Endocrine Laboratory. The endocrine samples were shipped as frozen aliquots of serum and were thawed only when the analyses were done. All samples were analyzed for each hormone in a single assay.

Serum concentrations of testosterone, estradiol, progesterone, and luteinizing hormone (LH) were measured using a double antibody radioimmunoassay. For LH, 200 uL canine serum was incubated in the presence of LH antibody (R-15) at a dilution of 1:40,000, for 24 h at 4 °C. Following incubation, 125I-LH was added to each tube and further incubated for 24 h at 4 °C. After 24 h, a secondary antibody was added to precipitate the immune complexes, and the reaction was incubated for 72 h at 4 °C. Following incubation, cold pour-off buffer was added, the tubes were centrifuged to pellet the immune complexes, and the pellet was counted in a gamma spectrophotometer. The detailed validation of this assay was reported by Nett [20].

Prior to analyses of testosterone, estradiol, and progesterone, serum samples were extracted with organic solvent and reconstituted in assay buffer to eliminate any matrix effects in the assay. Briefly, 200 uL reconstituted serum was incubated in the presence of I-125-labeled hormone and appropriate hormone antibody, at a dilution of 1:200,000, 1:400,000 and 1:48,000, respectively, for 6 h at 4 °C. After 6 h, a secondary antibody was added to precipitate the immune complexes, and the reaction was further incubated for 72 h at 4 °C. Following incubation, cold pour-off buffer was added, the tubes were centrifuged to pellet the immune complexes, and the pellet was counted in a gamma spectrophotometer. Please see Supplementary Methods for more information on assay methodology and intra-assay variation.

Cortisol, thyroid stimulating hormone (TSH) and thyroid hormone (T4) were measured by direct competitive inhibition ELISA. The ELISA kit for cortisol was purchased from CUSABIO (Houston, TX) and the ELISA kits for TSH and T4 were purchased from Biomatik (Wilmington, Delaware). For the measurement of TSH and T4, 50 uL sample or standard were added to each well along with 50 uL of analyte-specific antibody conjugate and incubated at 37°C for 60’ or 45’, respectively. Following incubation, the plate was washed three times with 200 uL wash buffer, 50 uL or 100 uL respectively of streptavidin-HRP conjugate was added to each well, and the plate was incubated for 30’ at 37 °C. After incubation, the plates were washed and 50 uL or 90 uL TMB substrate was added to each well and the plate was incubated in the dark at 37 °C for 15’ or 30’, respectively. Fifty microliters of stop solution were then added to each well and the absorbance was measured at 450 nm.

The protocol for measuring cortisol, by ELISA, was the same as the protocol for T4, except the sample or standard was incubated with antibody conjugate for 40’. In addition, an optical measurement at 540 nm was taken and subtracted from the 450 nm measurement to correct for optical imperfections in the plate.

### Data management and statistical analysis

Data were entered into Microsoft Excel spreadsheets and fully verified. Data were collated by treatment group and time point and summary parameters were calculated. A summary Zambelli Symptom Index was completed based on observations made between Day 0 to Day 90. Hormone concentrations below the assay’s detectable limit had a substitute value of 0 used for analyses and summaries. To assess the impact of the treatment levels, a two-way repeated measures analysis of variance was conducted. Data transformation was applied to variables as needed. The main between-subjects effect of the treatment group and the interaction of the treatment group and the within-subjects factor time (treatment x day) were evaluated. Statistical output was assessed via residual plots for method suitability. When appropriate, treatment means were compared using Tukey Pairwise Comparison Test, at *p* < 0.05. Statistical analyses were completed with Statistix 10.0 (Analytical Software 2013). Data were presented as the mean ± standard error.

## Results

All 12 dogs selected for participation completed the study. Adverse events during the treatment phase were rare and included one incident of a swollen digit of the paw (Group 1x) and one skin rash (Control Group), which were treated and resolved. One dog (Group 3x) had a seizure of moderate intensity on Day 45 and again on Day 87. Each time he was moved to a separate run for observation, treated with diazepam, and recovered. It was later noted that he had a seizure prior to enrollment in the study. The only other treatment during the study was for otitis in one dog in the Control Group.

Minimal variation was observed over time and between treatment groups for the behavioral scores, the Zambelli Scores, and the Body Condition scores. Thus, statistical analyses were not performed for these parameters. Behavioral observations during baseline were normal for all observations except on the first day (-8) when two dogs exhibited signs related to fear (urination, salivation). Only 30 of the 182 behavior observations conducted during the treatment phases indicated non-normal observations. Aggressive interactions were observed 9 times (5% of observations). This included 1 instance of aggressive play (Group 5x); 4 instances of non-contact aggression (barking or growling) (1 in Group 3x and 3 in Group 5x); and 4 instances of pinning/jumping/aggressive mounting another dog (2 in Group 1x, 1 in Group 3x, and 1 in Group 5x). Three of the five occurrences in Group 5x involved all group members. Aggressive interactions occurred only once within 3 days after the treatment when testosterone levels were expected to be higher. Twenty-one occurrences of diarrhea/loose feces or vomit in the enclosures were recorded across all groups and over time. These findings were common in the colony dogs especially when accessing the grass-covered exercise yards. There was no indication of symptoms of benign prostatic hyperplasia (as measured by the Zambelli Score) during the study. Body condition and waist circumference changed little over time.

Repeated measures analysis of variance results for clinical examination measures, including heart rate, temperature, and body weight, showed no significant differences between treatment groups or for the interaction between treatment group x day (*p* > 0.05). The analysis of respiration rate was not possible because 34% of the observations included missing data. This was due to the inability to measure respiration rate at the time of observation when the dog was panting.

Potassium levels were significantly higher for Group 5x than for Group 1x [F(3,8) = 6.27 *p* < 0.017]. The difference was evident across both baseline and treatment conditions, remained within the normal range for both groups, and was likely due to pre-existing biological differences rather than treatment effects. While the main treatment group effect was not significant for hemoglobin, the treatment group x day interaction was significant [F(15,40) = 2.26, *p* < 0.020], but no specific pairwise comparisons were significant. One dog in Group 5x had higher levels of hemoglobin than other dogs, but individual levels were generally consistent over time. No significant differences across treatment groups or the interaction between treatment group x day were found for any other hematology/biochemistry measurements (*p* > 0.05).

**Fig. 1:**
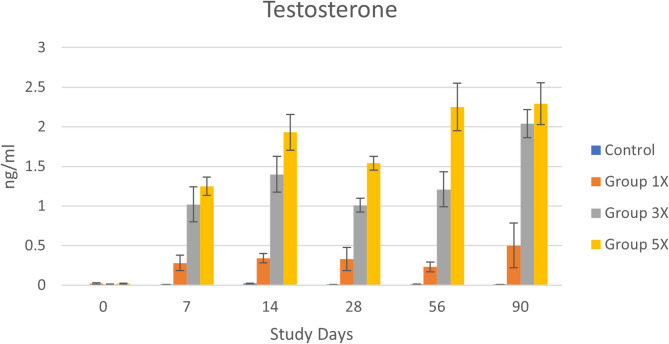
Testosterone levels (mean ± SE) across groups and days. Day 0 is untreated baseline for all groups. After baseline, Group 5x levels were significantly higher than other groups and Group 3x levels were significantly higher than Controls and Group 1x

Testosterone had a highly significant treatment group effect [F(3,8) = 131.7, *p* < 0.001] and for the interaction of treatment group x day [F(15,40) = 7.84, *p* < 0.001]. Testosterone levels at baseline were similarly low for all groups (average 0.01 ± 0.003 ng/ml), which was within the normal range for castrated dogs. After treatment, testosterone levels varied in a dose-dependent fashion, with Group 5x significantly higher than other groups, and Group 3x significantly higher than Controls and Group 1x. Testosterone levels for Group 1x were also higher than the control group, although the differences were not significant (Fig. 1). Testosterone levels for Group 3x and 5x increased to within the normal range for intact dogs (0.5–9.0 ng/ml) after treatment. Assessment of testosterone was conducted one week following each weekly treatment, so measurements likely reflect the lowest weekly level during each treatment period.

**Fig. 2:**
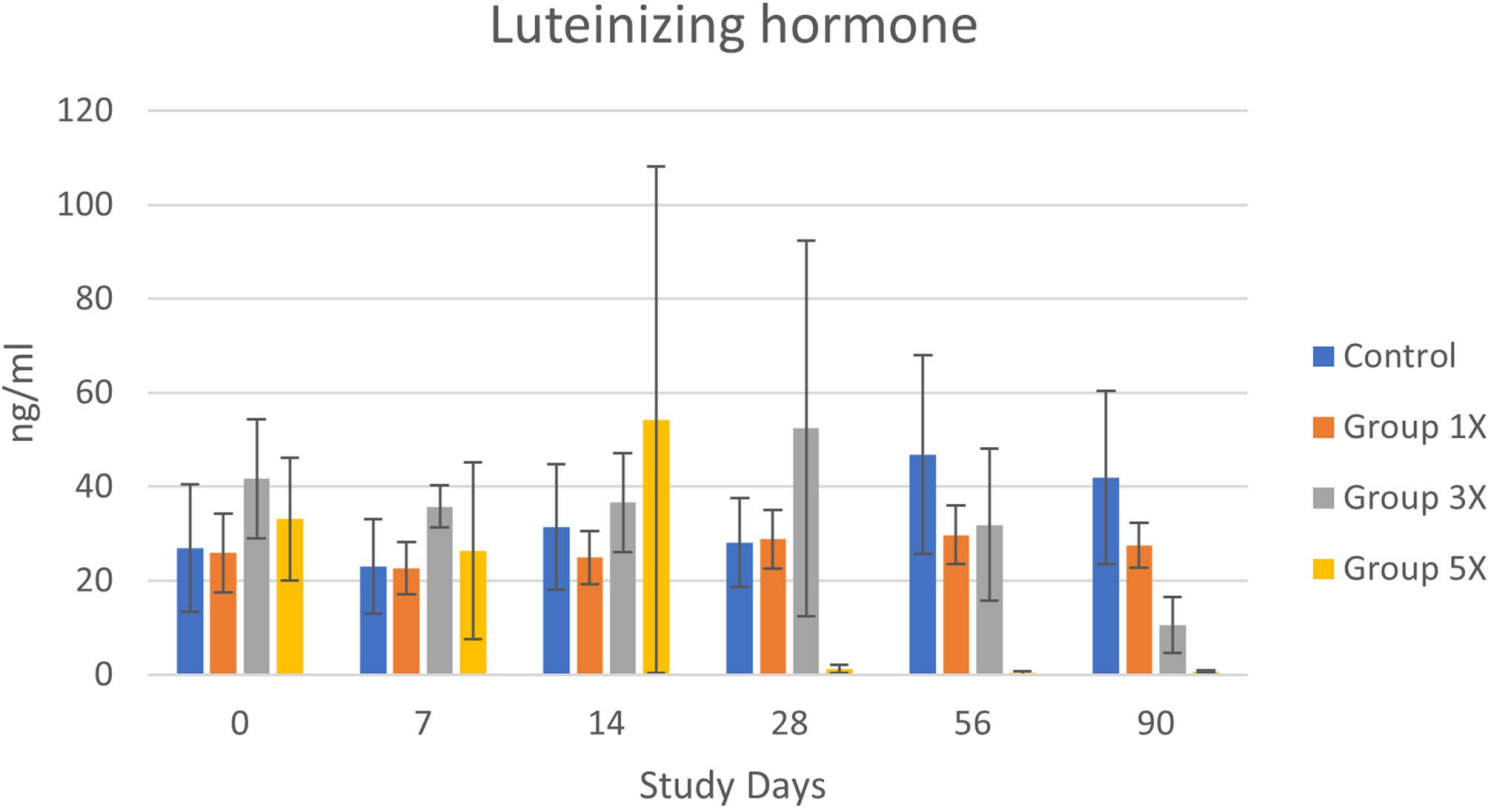
LH levels (mean ± SE) across groups and days. Day 0 is untreated baseline for all groups. Group 5x levels at days 28–90 were significantly lower than at day 0

Average luteinizing hormone levels were higher than the reference range for neutered males at baseline (31.87 ± 5.5 ng/ml, 11.4–65.8 ng/ml). Analysis of raw data indicated no significant differences between treatment groups for luteinizing hormone. However, visual evaluation revealed a notable decline for Group 3x and 5x over time (Fig. 2). Due to the presence of two extremely high outliers (> 130 ng/ml) in these treatment groups, the data were log transformed and reanalyzed. This data analysis revealed no significant difference between treatment groups at baseline, but an overall significant interaction between treatment group x day effect [F(15,40) = 2.58, *p* < 0.009]. This was due to Group 5x having significantly lower levels of LH at days 28, 56, and 90 compared to baseline. Group 3x also had a lower level by day 90, but this was not statistically significant.

**Fig. 3:**
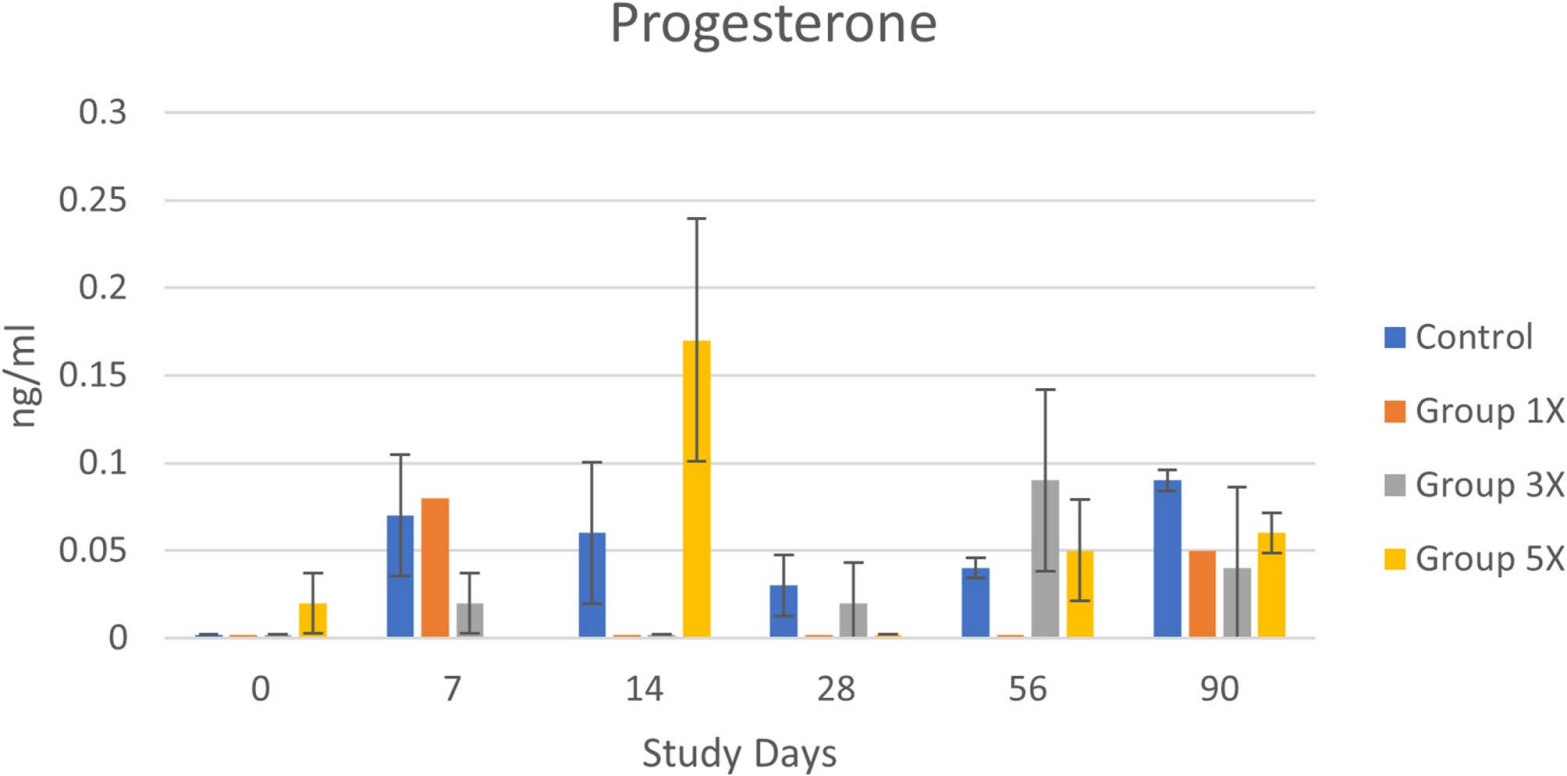
Progesterone levels (mean ± SE) across groups and days. Day 0 is untreated baseline for all groups. At day 14, Groups 1x and 3x levels were significantly lower than Group 5x

Analysis of variance indicated a significant interaction between treatment group x day for progesterone [F(15,40) = 2.59, *p* < 0.008]. Paired comparison testing indicated that this was due to a specific timepoint on day 14: Groups 1x and 3x had significantly lower progesterone levels (all samples 0 ng/ml) than Group 5x (mean 0.17 ng/ml). However, assessment and interpretation of progesterone levels were hampered by the high number of samples below the detectable limit (56.9%) as evidenced in Fig. 3. Analysis of estradiol also included a large number of samples with undetectable levels (70.8%) and no significant differences across groups. No significant differences were observed between treatment groups for cortisol, thyroid stimulating hormone, and thyroid hormone during the study.

## Discussion

The loss of gonadal hormones following spay or neuter has been linked to various health and behavior issues (for review see [3]). Treatment of spay-neuter syndrome through restoration of natural hormone levels in neutered male dogs is relatively new, with little scientific evaluation and a lack of established methods.

The current study provides the first safety and dosing information on injectable testosterone for the purpose of hormone restoration in castrated male dogs. The results support the safety of this method of treatment with few changes in the health parameters measured over 3 months. Findings did reflect the endocrine changes expected after supplementation of testosterone, including increased testosterone levels and some decline in LH, without significant side effects over three months, even for dogs provided five times the standard dose.

Testosterone dosing level and administration details are not standardized for dogs and multiple delivery methods for testosterone exist (see Supplemental Table 4). Previous canine research on testosterone administration is very limited and includes a study inducing benign prostatic hyperplasia in neutered dogs given testosterone-filled implants delivering a dose of 0.25 mg/kg/day [21]. A second publication of a retrospective evaluation of testosterone therapy to treat incontinence in castrated male dogs found no side effects for monthly intramuscular injection of testosterone cypionate (median dose 1.5 mg/kg/month) [16]. Human research reports that weekly or bi-weekly subcutaneous injections provide more stable levels of testosterone than injections of longer duration and produce less pain than the intramuscular route [22]. This finding was confirmed in a single dog case study using subcutaneous testosterone cypionate at 0.5 mg/kg/week to treat spay-neuter syndrome [17]. Building on the success of this study, the same dose and delivery schedule were used in the current canine target safety study.

Few unexpected changes in health and behavior were noted during the three-month study. Three adverse events occurred but did not appear to be related to treatment. Two were minor health issues (swollen digit and rash) which were treated and recovered. Previous canine research found a lack of adverse events with monthly and weekly doses of testosterone cypionate [16, 17].

The third adverse event involved a beagle receiving three times the normal dose that had two seizures during the study, after which he recovered uneventfully. A record review found that he also had a seizure prior to the study, indicating that he may have idiopathic epilepsy. This condition has a genetic basis and has a high occurrence in beagles [23]. A complex relationship exists between reproductive hormones and epilepsy. Research in humans and mice reported that testosterone may be metabolized to dihydrotestosterone that can have anticonvulsant effects, or to estradiol that can have pro-convulsant effects [24, 25]. Increased estradiol was not detected in the study dog and no other measures provided a reason for the seizures, which could have been related to a number of environmental, social, or individual factors. However, given that conclusions on the effects of testosterone on canine epilepsy are inconclusive at present [26], careful consideration needs to be given before testosterone therapy is provided for a dog with a history of seizures.

Body condition is known to influence reproductive hormone levels in humans. For example, most men with obesity and higher waist circumference have decreased testosterone levels, dysfunction of the hypothalamic–pituitary–testicular axis, and increased estradiol levels due to aromatization of testosterone in adipose tissue [27, 28]. Testosterone therapy reduces fat mass and improves adipose tissue function [27], and has been recommended for obese hypogonadal men [29]. In our study, the dogs’ body condition scores were in the ideal range and body weight, waist circumference, and body condition scores were stable throughout the study. Thus, significant aromatization of testosterone in adipose tissue was unlikely in this study population, and no changes in estradiol were found over the three month study. It is possible that treating obese dogs with testosterone could result in increased estradiol levels but may also result in improved body condition over time.

Behavior records conducted twice daily indicated very few abnormal observations. Gastrointestinal upset (vomiting, diarrhea) was considered normal for the colony and related to access to the grassy play areas. Aggressive interactions between the group-living dogs were rare and non-injurious. Five of the nine aggressive incidences occurred in Group 5x while none occurred in the Control Group. However, conclusions about a treatment effect were not possible due to the low incidence of aggression, small number of subjects, and other uncontrolled factors influencing behavior (e.g., individual differences, dominance/social group changes, environmental factors). A more rigorous behavior study would be required to fully understand the relationship between canine testosterone therapy and behavior.

The goal of testosterone restoration is to maintain the serum testosterone level within the normal range for intact dogs to bring the endocrine system back into balance. Treatment with exogenous testosterone was effective at altering the hormone profile of the dogs in this study. As expected, testosterone levels increased after treatment in a dose dependent fashion, with dogs given 3–5 times the standard dose reaching the normal testosterone range for intact dogs. The dogs receiving the 1x standard dose of 0.5 mg/kg/week had a slight increase in testosterone levels. Because blood measurements in this study were taken one week after injection, it is likely that levels were higher mid-week. A previous case study found that weekly injections of the standard dose maintained testosterone levels at 1.2–2.2 ng/mL when measured three days after dosing [17]. Pharmacokinetic studies would be needed to clarify the metabolism of testosterone cypionate in dogs.

In the adult testicles of most animals, the Leydig cells express aromatase and produce estradiol more than the Sertoli cells [30]. Thus, elevated estradiol levels are a risk of testosterone therapy in men, with as many as 20% having high estradiol levels [31]. The adrenal glands also produce low levels of estradiol, which occurs even in castrated dogs [32]. In our study, there was no significant relationship between estradiol levels and treatment group, with most dogs having undetectable levels. An ACTH stimulation test may be helpful in detecting any changes in adrenal estradiol production with testosterone restoration, but some studies have reported no difference between intact and castrated male dogs [33, 34].

We also found no significant differences between treatment groups for cortisol, thyroid stimulating hormone, or thyroid hormone during the study. While some studies indicate a role for crosstalk between testosterone and thyroid hormones, our results reflect previous research showing no relationship between thyroid function and gonadectomy in dogs [35]. A significant difference was found between groups for progesterone at one time point, but interpretation was limited due to small sample size and values below the limit of detection.

Neutered dogs often have supraphysiologic levels of luteinizing hormone due to the lack of negative feedback of testosterone on the pituitary gland. Given the presence of LH receptors throughout the canine body, there is a possible relationship between gonadectomy, high luteinizing hormone and spay-neuter syndrome [4]. In a previous case study, LH levels were reduced somewhat with testosterone therapy, but a gonadotropin-releasing hormone (GnRH) agonist was required to bring supraphysiologic levels back to normal [17]. In the current study, most dogs had supraphysiologic levels of LH at baseline. Only those receiving five times the standard dose of testosterone had significantly lower levels of LH compared to baseline starting at Day 28 of the study. Our study confirms that LH is responsive to testosterone supplementation, but a return to normal levels only occurred at a high testosterone dose. The focus of the current study was on restoration of testosterone to intact levels in neutered dogs, but LH levels should be measured before and after testosterone therapy to determine if a GnRH agonist may still be needed to reduce LH. Deslorelin implants are commercially available GnRH agonists, marketed as Suprelorin^®^ for nonsurgical fertility control. The implants provide prolonged stimulation of GnRH receptors, leading to their desensitization of the receptors and a reduction of LH resulting in lack of sperm production in intact dogs [36].

This study provided the first basic safety information on testosterone therapy for neutered dogs, but it did not evaluate the effects of long-term administration of testosterone. Two small studies provide initial evidence for long-term safety outcomes. A previous study on testosterone cypionate given at 3–6 week intervals reported no adverse outcomes, including one dog on the therapy for 19 months [16]. A single dog on the same weekly dose as the current study continued to have positive effects and no adverse events for 20 months of the study [17] and has remained on the therapy for over 4.5 years (L. Brent, pers. comm. 2024-10-01).

Testosterone therapy has known risks in humans and other animals and should not be used in dogs with serious health conditions (e.g., hepatic, renal, prostate, heart disease). To maintain good health during hormone therapy, routine clinical assessment and hematology/biochemistry evaluation is important prior to and at regular intervals. Measurement of testosterone and LH levels is also helpful before and during treatment.

The type, dosing, and timing of testosterone administration is also important. Oral administration of C-17α alkylated testosterones is well known to cause liver damage in dogs and humans [37, 38] and is not recommended for testosterone replacement. Other forms of testosterone have not been reported to have the same liver toxicity effects in humans, but caution should be used if considering testosterone therapy in a dog with liver disease or a poorly functioning liver. Dogs have fluctuating levels of testosterone over the course of 24 h [39–41] which cannot be replicated with current methods of testosterone replacement. Daily transdermal applications would provide the most stable testosterone levels but lack of application sites, possible ingestion, and transfer to humans make this route unfeasible (Supplemental Table 4). Subcutaneous injectable testosterone esters with frequent dosing (once or twice per week) provide more stable levels as compared to monthly doses, less pain, and reduce the incidence of erythrocytosis in men [42].

Testosterone restoration in neutered dogs has the potential to increase the risk of hormone-dependent disease processes, such as benign prostatic hyperplasia (BPH). High levels of testosterone cypionate and testosterone propionate have experimentally induced BPH in dogs [21, 43]. The current study used a lower dose of testosterone and did not find any symptoms of BPH as measured by the Zambelli Score for any treatment group. This finding could be due to treatment duration, subject age, age of castration, testosterone delivery, or the method used to assess BPH compared to the previous studies. However, a long-term study reported that only half of the dogs castrated at 6 or 12 months of age then given testosterone and estrogen replacement developed BPH at 5 years of age, compared to 100% of the intact controls [44]. All dogs exhibited some atrophy of the prostate, but the testes were important to the full development of BPH [44]. These findings indicate that BPH may be less problematic for dogs treated with hormone restoration, but regular assessment of the prostate before and during testosterone therapy is recommended.

Secondary erythrocytosis (polycythemia) causing higher hemoglobin or hematocrit levels in men is the most common side effects of testosterone therapy, although evidence of harm is scarce [22, 45, 46]. In humans, an increased duration of high testosterone levels results in an increased risk of erythrocytosis, which is more common with infrequent intramuscular injection and pellet delivery [46]. For example, bi-weekly dosing resulting in higher average testosterone levels in men was associated with a greater risk of erythrocytosis as compared to weekly dosing [47]. Endocrinopathy-associated erythrocytosis can result from hormones like testosterone that stimulate erythropoiesis in animals [48]. In this study, hemoglobin and hematocrit levels of the dogs stayed within the normal ranges. While a significant interaction between treatment group and day was found for hemoglobin, no specific comparisons were significant and individual levels were highly consistent over time. Red blood cell count and hematocrit did not differ by treatment group or across time. As recommended for humans, hemoglobin and hematocrit should be evaluated regularly for dogs on testosterone therapy.

This target safety study of testosterone therapy in dogs provided valuable information but had limitations. The small group sizes hindered some statistical analyses and conclusions for several variables and disallowed consideration of breed or size effects. Evaluation of potential prostate enlargement due to testosterone restoration would be enhanced with prostatic ultrasound, longer study duration, and additional measures (e.g., dihydrotestosterone). As hormone restoration is a new canine treatment area, future research would be needed to compare different delivery methods and doses, long-term outcomes, behavioral measures, and how testosterone is metabolized over time.

## Conclusion

This was the first target safety study providing information for testosterone restoration in neutered dogs. Weekly subcutaneous dosing was effective and safe even at five times the standard dose in the 3-month study. Only one dog had a significant adverse event, which appeared to be related to a prior history of idiopathic epilepsy. Few changes in health and behavior parameters were noted across subjects. No evidence of typical risks for testosterone therapy, such as benign prostatic hyperplasia or erythrocytosis, were found. The safety and tolerability of weekly subcutaneous (as opposed to less frequent intramuscular) testosterone delivery reported in human studies [42] were confirmed in this study.

The dose used in the study (0.5 mg/kg/wk) was effective at increasing testosterone levels. If a reduction is desired in the high LH levels that are common in neutered dogs, additional treatment (e.g., GnRH agonist) is necessary, as our study indicated that testosterone only significantly decreased LH levels in those dogs receiving five times the standard dose. Additional long-term studies with more subjects will further clarify statistical outcomes and allow consideration of other factors such as breed, age, and size of the dog.

The information from this report should assist veterinarians in determining if testosterone restoration is appropriate for treating dogs with signs of spay-neuter syndrome and provides basic dosing and safety data necessary for its use.

## Electronic supplementary material

Below is the link to the electronic supplementary material.

Supplementary Material 1

## Data Availability

Data are provided within the manuscript or supplementary information files.

## Abbreviations

ALT: Alanine aminotransferase
ALP: Alkaline Phosphatase
BUN: Urea nitrogen
CBC: Complete blood count
ELISA: Enzyme-linked immunosorbent assay
FSH: Follicle stimulating hormone
GnRH: Gonadotropin releasing hormone
Hb: Hemoglobin
LH: Luteinizing hormone
RBC: Red blood count
SE: Standard error
T4: Thyroid hormone
TSH: Thyroid stimulating hormone
WBC: White blood count
