# Supplementary material for: Safety and dosing of testosterone for hormone restoration in neutered dogs
Source: BMC Vet Res. 2025 Jul 9;21:449. doi: 10.1186/s12917-025-04869-8 (PMC12239267; doi:10.1186/s12917-025-04869-8)
Supplement: Supplementary file 1 — Supplementary Material 1 [file 12917_2025_4869_MOESM1_ESM.docx]

**Supplementary Material**

Supplementary Methods: Blood and hormone analysis

The assay used for canine LH has been used by the Colorado State University Endocrine Laboratory for the past 50 years and has not changed except for canine LH standard that has increased in purity over time. It is a heterologous assay using an antibody to ovine LH, radioiodinated ovine LH and purified canine LH as standard. This antibody (GDN-15) cross-reacts with LH from numerous species including ovine, bovine, equine, canine, feline, rat, mouse, dolphin, elephant, monkey, human, and porcine. The antibody binds a fragment of LH that is highly conserved across species.

Estradiol, testosterone, and progesterone assays were also conducted utilizing reagents produced by the Colorado State University Endocrine Laboratory over 50 years ago and are validated for use in numerous species [(1-3)].

Since the steroids were removed from serum via organic extraction prior to analysis, any matrix effects due to uniqueness of canine serum were eliminated. The average intra-assay variation was calculated at a point approximating 80% and 30% on the standard curve for each of the assays and is included in the table below. The variation was less than 6% in each assay.

Supplementary Table Methods: Intra-assay variation of hormone assays.

|  | **Testosterone** | |  | **Luteinizing Hormone** | |  | **Estrogen** | |  | **Progesterone** | |
| --- | --- | --- | --- | --- | --- | --- | --- | --- | --- | --- | --- |
| **Dose** | 400 pg | 10 pg |  | 4 ng | 1 ng |  | 16 pg | 1.02 pg |  | 1 ng | 64 pg |
| **Curve 1** | 33.90 | 82.90 |  | 28.40 | 66.40 |  | 27.7 | 68.7 |  | 23.00 | 70.70 |
| **Curve 2** | 32.10 | 78.10 |  | 26.20 | 61.50 |  | 29.00 | 69.9 |  | 22.20 | 67.30 |
| **Mean** | 33.00 | 80.50 |  | 27.30 | 63.95 |  | 28.35 | 69.3 |  | 22.60 | 69.00 |
| **SD** | 1.27 | 3.39 |  | 1.56 | 3.46 |  | 0.92 | 0.85 |  | 0.57 | 2.40 |
| **%CV** | 3.86% | 4.22% |  | 5.70% | 5.42% |  | 3.24% | 1.22% |  | 2.50% | 3.48% |
| **Ave %CV** |  | 4.04% |  |  | 5.56% |  |  | 2.23% |  |  | 2.99% |

Supplementary Table 1: Clinical examination parameters. Group means ± 1 standard deviation.

| Study Day | Control | Group 1x | Group 3x | Group 5x |
| --- | --- | --- | --- | --- |
| Heart Rate (beats/minute) | | | | |
| 0 | 83.3 ± 15.3 | 76.7 ± 2.9 | 86.0 ± 6.9 | 92.7 ± 23.7 |
| 7 | 91.7 ± 17.6 | 96.7 ± 5.8 | 106.7 ± 11.5 | 93.3 ± 15.3 |
| 14 | 86.7 ± 25.2 | 86.7 ± 5.8 | 100.0 ± 10.0 | 96.7 ± 11.5 |
| 21 | 93.3 ± 5.8 | 83.3 ± 11.5 | 83.3 ± 11.5 | 83.3 ± 11.5 |
| 28 | 80.7 ± 5.1 | 78.3 ± 12.6 | 87.3 ± 6.4 | 103.3 ± 20.8 |
| 35 | 90.0 ± 10.0 | 88.3 ± 2.9 | 95.0 ± 5.0 | 93.3 ± 11.5 |
| 42 | 91.7 ± 2.9 | 83.3 ± 5.8 | 94.3 ± 6.0 | 92.7 ± 4.0 |
| 49 | 88.3 ± 2.9 | 86.7 ± 5.8 | 96.7 ± 5.8 | 92.7 ± 6.4 |
| 56 | 80.7 ± 9.0 | 88.7 ± 1.2 | 93.0 ± 8.2 | 90.3 ± 5.7 |
| 63 | 83.3 ± 7.0 | 96.0 ± 4.0 | 92.0 ± 6.9 | 96.0 ± 22.3 |
| 70 | 95.0 ± 13.2 | 98.3 ± 12.6 | 100.0 ± 5.0 | 100.0 ± 10.0 |
| 77 | 90.0 ± 34.6 | 100.0 ± 10.0 | 103.3 ± 15.3 | 113.3 ± 15.3 |
| 84 | 80.0 ± 17.3 | 80.3 ± 4.5 | 89.3 ± 10.1 | 97.3 ± 28.4 |
| 90 | 91.7 ± 16.1 | 91.7 ± 7.6 | 93.3 ± 10.4 | 95.0 ± 5.0 |
| Respiration Rate (breaths/minute) | | | | |
| 0 | 30.0 ± 0.0 | 24.7 ± 4.2 | 25.0 ± 0.0 | 31.0 ± 0.3 |
| 7 | 20.0 ± 0.0 | 27.0 ± 1.7 | 26.5 ± 2.1 | 27.5 ± 0.1 |
| 14 | 30.0 ± 0.0 | 23.0 ± 0.0 | 28.0 ± 0.0 | 28.0 ± 0.4 |
| 21 | 21.0 ± 0.0 | 22.5 ± 3.5 | 25.0 ± 0.0 | 30.0 ± 0.3 |
| 28 | 25.5 ± 0.7 | 26.0 ± 3.5 | 30.0 ± 0.0 | 30.0 ± 0.8 |
| 35 | 26.0 ± 2.8 | 24.7 ± 3.1 | 34.0 ± 8.5 | 27.0 ± 0.4 |
| 42 | 26.7 ± 3.1 | 24.0 ± 0.0 | 37.3 ± 19.7 | 23.3 ± 0.2 |
| 49 | --- | 26.0 ± 3.5 | 30.0 ± 0.0 | 30.0 ± 0.6 |
| 56 | 24.7 ± 1.2 | 24.7 ± 1.2 | 31.0 ± 1.4 | 24.0 ± 0.4 |
| 63 | --- | 24.0 ± 0.0 | 24.0 ± 0.0 | 28.0 ± 0.8 |
| 70 | 35.0 ± 7.1 | 30.3 ± 4.0 | 35.0 ± 7.1 | 45.0 ± 0.4 |
| 77 | 26.5 ± 2.1 | 37.3 ± 4.6 | --- | 43.0 ± 0.4 |
| 84 | 33.0 ± 6.2 | 20.0 ± 5.7 | 29.3 ± 12.9 | 36.0 ± 0.2 |
| 90 | 31.5 ± 4.9 | 26.7 ± 4.2 | 32.5 ± 0.7 | 30.0 ± 0.6 |
| Rectal Temperature (°C) | | | | |
| 0 | 38.6 ± 0.2 | 38.4 ± 0.1 | 38.1 ± 0.2 | 38.4 ± 0.3 |
| 7 | 38.4 ± 0.2 | 38.4 ± 0.3 | 38.4 ± 0.3 | 38.7 ± 0.1 |
| 14 | 38.6 ± 0.2 | 38.4 ± 0.3 | 38.3 ± 0.2 | 38.7 ± 0.4 |
| 21 | 38.4 ± 0.2 | 38.6 ± 0.5 | 38.4 ± 0.3 | 38.7 ± 0.3 |
| 28 | 38.4 ± 0.3 | 38.5 ± 0.2 | 38.2 ± 0.2 | 38.2 ± 0.8 |
| 35 | 38.1 ± 0.2 | 38.2 ± 0.3 | 38.2 ± 0.5 | 38.3 ± 0.4 |
| 42 | 38.5 ± 0.4 | 38.4 ± 0.5 | 38.4 ± 0.2 | 38.5 ± 0.2 |
| 49 | 38.2 ± 0.1 | 38.4 ± 0.4 | 38.1 ± 0.4 | 38.3 ± 0.6 |
| 56 | 38.2 ± 0.4 | 38.4 ± 0.6 | 38.3 ± 0.4 | 38.2 ± 0.4 |
| 63 | 38.4 ± 0.4 | 38.4 ± 0.4 | 38.7 ± 0.3 | 38.6 ± 0.8 |
| 70 | 38.6 ± 0.2 | 38.3 ± 0.4 | 38.1 ± 0.2 | 38.7 ± 0.4 |
| 77 | 38.6 ± 0.3 | 38.3 ± 0.5 | 38.0 ± 0.2 | 38.4 ± 0.4 |
| 84 | 38.3 ± 0.4 | 38.5 ± 0.3 | 38.3 ± 0.2 | 38.3 ± 0.2 |
| 90 | 38.7 ± 0.2 | 38.2 ± 0.4 | 38.0 ± 0.1 | 38.0 ± 0.6 |
| Bodyweight (kg) | | | | |
| 0 | 29.8 ± 11.6 | 30.1 ± 13.0 | 24.2 ± 10.3 | 21.6 ± 7.6 |
| 7 | 29.9 ± 11.4 | 30.8 ± 13.3 | 24.4 ± 10.2 | 22.1 ± 8.4 |
| 14 | 29.3 ± 11.5 | 30.2 ± 12.7 | 24.3 ± 9.4 | 22.0 ± 8.4 |
| 21 | 29.3 ± 11.3 | 30.6 ± 13.1 | 24.0 ± 8.8 | 22.3 ± 8.8 |
| 28 | 29.2 ± 11.4 | 30.2 ± 13.5 | 24.5 ± 9.2 | 22.2 ± 8.1 |
| 35 | 29.8 ± 11.7 | 30.5 ± 12.4 | 24.7 ± 9.0 | 23.0 ± 9.2 |
| 42 | 29.3 ± 11.7 | 30.2 ± 12.5 | 24.2 ± 8.5 | 22.6 ± 9.3 |
| 49 | 29.1 ± 11.8 | 30.4 ± 12.7 | 24.2 ± 9.0 | 22.7 ± 9.3 |
| 56 | 28.9 ± 11.7 | 30.1 ± 12.5 | 24.6 ± 9.5 | 22.9 ± 9.4 |
| 63 | 28.9 ± 11.8 | 30.4 ± 12.5 | 24.2 ± 8.5 | 22.7 ± 9.3 |
| 70 | 29.2 ± 11.9 | 30.6 ± 12.5 | 24.7 ± 8.2 | 22.9 ± 9.4 |
| 77 | 29.4 ± 12.3 | 31.0 ± 12.5 | 25.3 ± 8.4 | 23.5 ± 9.7 |
| 84 | 29.2 ± 11.6 | 30.4 ± 12.7 | 25.4 ± 8.5 | 23.3 ± 10.0 |
| 90 | 28.7 ± 12.2 | 30.2 ± 12.6 | 25.1 ± 8.4 | 23.1 ± 10.0 |

Supplementary Table 2: Clinical pathology parameters. Group means ± 1 standard deviation. Reference range is listed for each parameter.

| Study Day | Control | Group 1x | Group 3x | Group 5x |
| --- | --- | --- | --- | --- |
| White Blood Cells (x 10^9^/L) (5.3 - 14.9) | | | | |
| 0 | 12.6 ± 4.71 | 11.8 ± 2.40 | 13.3 ± 0.55 | 15.5 ± 2.62 |
| 7 | 12.9 ± 1.77 | 13.8 ± 1.17 | 15.8 ± 2.46 | 13.5 ± 3.56 |
| 14 | 12.5 ± 2.77 | 12.9 ± 1.60 | 15.0 ± 1.67 | 12.3 ± 3.27 |
| 28 | 11.7 ± 1.88 | 12.4 ± 0.65 | 13.4 ± 1.02 | 13.6 ± 1.74 |
| 56 | 12.1 ± 1.50 | 13.9 ± 1.27 | 14.3 ± 2.44 | 13.1 ± 3.40 |
| 90 | 13.9 ± 1.01 | 12.6 ± 0.21 | 13.8 ± 2.10 | 12.3 ± 4.04 |
| Hemoglobin (g/L) (130 - 200) | | | | |
| 0 | 149.3 ± 18.3 | 142.7 ± 14.5 | 140.0 ± 9.0 | 159.7 ± 18.1 |
| 7 | 142.3 ± 21.2 | 142.7 ± 9.5 | 144.7 ± 7.5 | 153.0 ± 23.4 |
| 14 | 139.7 ± 12.2 | 137.0 ± 10.0 | 143.7 ± 2.1 | 151.3 ± 16.2 |
| 28 | 140.0 ± 13.2 | 138.7 ± 12.5 | 150.3 ± 2.3 | 157.3 ± 24.8 |
| 56 | 137.0 ± 13.1 | 140.7 ± 14.0 | 144.3 ± 6.0 | 157.0 ± 26.3 |
| 90 | 134.0 ± 11.4 | 151.3 ± 5.1 | 149.3 ± 3.8 | 164.3 ± 30.0 |
| Red Blood Cells (x 10^12^/L) (5.5 - 8.4) | | | | |
| 0 | 6.3 ± 0.64 | 6.7 ± 0.96 | 5.9 ± 0.47 | 6.7 ± 0.81 |
| 7 | 5.9 ± 0.78 | 6.7 ± 0.96 | 6.1 ± 0.36 | 6.5 ± 1.03 |
| 14 | 5.8 ± 0.35 | 6.5 ± 1.00 | 6.0 ± 0.12 | 6.3 ± 0.56 |
| 28 | 5.8 ± 0.45 | 6.4 ± 0.97 | 6.2 ± 0.12 | 6.5 ± 0.95 |
| 56 | 5.7 ± 0.60 | 6.6 ± 0.96 | 6.1 ± 0.29 | 6.6 ± 0.98 |
| 90 | 5.6 ± 0.26 | 7.0 ± 0.79 | 6.3 ± 0.15 | 6.9 ± 1.18 |
| Hematocrit (L/L) (0.37 - 0.55) | | | | |
| 0 | 0.44 ± 0.05 | 0.42 ± 0.03 | 0.41 ± 0.03 | 0.46 ± 0.05 |
| 7 | 0.41 ± 0.05 | 0.43 ± 0.02 | 0.43 ± 0.02 | 0.45 ± 0.06 |
| 14 | 0.41 ± 0.03 | 0.42 ± 0.02 | 0.42 ± 0.01 | 0.44 ± 0.04 |
| 28 | 0.42 ± 0.03 | 0.42 ± 0.03 | 0.44 ± 0.00 | 0.46 ± 0.06 |
| 56 | 0.40 ± 0.04 | 0.42 ± 0.03 | 0.42 ± 0.01 | 0.45 ± 0.06 |
| 90 | 0.40 ± 0.03 | 0.46 ± 0.01 | 0.43 ± 0.01 | 0.47 ± 0.07 |
| Alkaline Phosphatase (U/L) (1 - 182) | | | | |
| 0 | 43.0 ± 18.2 | 43.0 ± 28.8 | 38.0 ± 17.7 | 45.7 ± 25.5 |
| 7 | 40.3 ± 19.9 | 50.0 ± 32.9 | 40.0 ± 21.1 | 46.3 ± 26.3 |
| 14 | 45.7 ± 36.0 | 42.7 ± 28.5 | 40.3 ± 23.1 | 50.3 ± 24.8 |
| 28 | 37.3 ± 17.9 | 40.3 ± 26.1 | 42.3 ± 23.5 | 44.0 ± 21.6 |
| 56 | 32.7 ± 15.0 | 36.3 ± 17.4 | 32.3 ± 11.9 | 36.0 ± 13.1 |
| 90 | 26.0 ± 12.2 | 32.7 ± 14.4 | 31.3 ± 9.9 | 27.3 ± 7.5 |
| Alanine Aminotransferase (U/L) (1 - 80) | | | | |
| 0 | 33.7 ± 5.9 | 32.0 ± 4.0 | 30.0 ± 6.2 | 29.3 ± 5.9 |
| 7 | 32.3 ± 6.4 | 32.3 ± 3.8 | 40.3 ± 19.6 | 26.3 ± 4.0 |
| 14 | 30.7 ± 6.0 | 25.7 ± 4.6 | 30.0 ± 6.1 | 26.0 ± 4.4 |
| 28 | 40.0 ± 13.1 | 28.7 ± 6.1 | 43.7 ± 22.5 | 32.0 ± 6.9 |
| 56 | 30.7 ± 5.0 | 26.0 ± 2.0 | 29.0 ± 6.1 | 35.0 ± 13.1 |
| 90 | 31.0 ± 4.6 | 31.3 ± 8.1 | 37.3 ± 3.2 | 36.7 ± 7.4 |
| Urea (mmol/L) (2.5 - 9.0) | | | | |
| 0 | 4.87 ± 0.49 | 4.73 ± 1.46 | 4.23 ± 1.02 | 4.70 ± 0.40 |
| 7 | 5.23 ± 0.47 | 5.60 ± 2.15 | 3.70 ± 0.75 | 3.93 ± 0.25 |
| 14 | 5.03 ± 0.65 | 4.43 ± 1.17 | 4.07 ± 0.38 | 4.03 ± 0.21 |
| 28 | 4.70 ± 0.44 | 4.30 ± 0.87 | 3.30 ± 0.53 | 3.97 ± 0.21 |
| 56 | 4.50 ± 0.46 | 4.47 ± 1.36 | 3.73 ± 0.64 | 4.53 ± 0.75 |
| 90 | 4.27 ± 0.25 | 4.10 ± 0.82 | 3.57 ± 0.76 | 5.00 ± 0.75 |
| Creatinine (µmol/L) (44 - 125) | | | | |
| 0 | 88.7 ± 4.2 | 82.7 ± 5.5 | 73.0 ± 10.8 | 87.0 ± 7.0 |
| 7 | 79.0 ± 7.5 | 77.7 ± 7.0 | 68.0 ± 8.2 | 77.0 ± 1.0 |
| 14 | 83.7 ± 6.4 | 79.0 ± 8.2 | 76.0 ± 12.5 | 79.7 ± 5.7 |
| 28 | 84.7 ± 6.5 | 82.0 ± 7.8 | 74.0 ± 11.5 | 80.0 ± 4.4 |
| 56 | 85.0 ± 3.6 | 78.3 ± 10.1 | 70.0 ± 12.1 | 76.3 ± 0.6 |
| 90 | 82.0 ± 5.6 | 78.3 ± 6.7 | 72.0 ± 16.5 | 85.7 ± 9.9 |
| Protein (g/L) (53 - 73) | | | | |
| 0 | 64.0 ± 2.65 | 64.3 ± 3.06 | 64.0 ± 3.46 | 64.0 ± 2.65 |
| 7 | 63.3 ± 5.86 | 65.0 ± 3.00 | 68.3 ± 5.51 | 66.0 ± 6.24 |
| 14 | 64.7 ± 4.93 | 66.3 ± 1.53 | 68.7 ± 5.03 | 65.3 ± 4.62 |
| 28 | 63.0 ± 4.58 | 67.3 ± 3.51 | 68.7 ± 5.51 | 67.0 ± 6.00 |
| 56 | 71.0 ± 5.20 | 69.3 ± 5.03 | 68.7 ± 10.02 | 68.0 ± 2.65 |
| 90 | 76.7 ± 12.34 | 71.0 ± 4.58 | 73.0 ± 3.61 | 70.0 ± 4.36 |
| Sodium (mmol/L) (140 - 155) | | | | |
| 0 | 145 ± 1.15 | 146 ± 1.53 | 145 ± 1.73 | 145 ± 1.00 |
| 7 | 145 ± 0.58 | 146 ± 2.52 | 145 ± 1.00 | 145 ± 1.00 |
| 14 | 144 ± 2.89 | 145 ± 0.58 | 146 ± 0.00 | 144 ± 1.15 |
| 28 | 145 ± 1.73 | 146 ± 1.00 | 145 ± 1.00 | 145 ± 0.58 |
| 56 | 145 ± 0.58 | 146 ± 0.00 | 147 ± 0.58 | 146 ± 0.00 |
| 90 | 144 ± 1.00 | 145 ± 0.58 | 146 ± 0.58 | 146 ± 0.58 |
| Potassium (mmol/L) (3.8 - 5.8) | | | | |
| 0 | 5.00 ± 0.30 | 4.60 ± 0.10 | 4.77 ± 0.15 | 5.17 ± 0.21 |
| 7 | 4.87 ± 0.23 | 4.77 ± 0.29 | 4.87 ± 0.15 | 5.10 ± 0.10 |
| 14 | 4.83 ± 0.06 | 4.63 ± 0.25 | 4.83 ± 0.21 | 5.07 ± 0.12 |
| 28 | 5.27 ± 0.38 | 4.93 ± 0.21 | 5.37 ± 0.06 | 5.57 ± 0.23 |
| 56 | 4.87 ± 0.25 | 4.67 ± 0.15 | 4.73 ± 0.12 | 5.17 ± 0.25 |
| 90 | 4.93 ± 0.23 | 4.67 ± 0.06 | 4.73 ± 0.15 | 4.87 ± 0.25 |
| Glucose (mmol/L) (3.5 - 6.7) | | | | |
| 0 | 2.93 ± 0.55 | 2.97 ± 0.38 | 2.87 ± 0.12 | 2.10 ± 0.17 |
| 7 | 2.33 ± 0.47 | 1.90 ± 0.36 | 2.07 ± 0.45 | 1.97 ± 0.78 |
| 14 | 3.10 ± 0.35 | 2.23 ± 0.32 | 1.70 ± 0.46 | 2.03 ± 0.38 |
| 28 | 1.97 ± 0.50 | 1.87 ± 0.75 | 1.77 ± 0.12 | 1.77 ± 0.15 |
| 56 | 1.80 ± 0.10 | 1.30 ± 0.70 | 1.60 ± 0.40 | 1.17 ± 0.12 |
| 90 | 2.53 ± 0.32 | 2.37 ± 0.21 | 2.07 ± 0.49 | 1.87 ± 0.78 |

Supplementary Table 3: Endocrine parameters. Group means ± 1 standard deviation.

| Study Day | Control | Group 1x | Group 3x | Group 5x |
| --- | --- | --- | --- | --- |
| Progesterone (ng/mL) | | | | |
| 0 | 0.00 ± 0.00 | 0.00 ± 0.00 | 0.00 ± 0.00 | 0.02 ± 0.03 |
| 7 | 0.07 ± 0.06 | 0.08 ± 0.04 | 0.02 ± 0.03 | 0.00 ± 0.00 |
| 14 | 0.06 ± 0.07 | 0.00 ± 0.00 | 0.00 ± 0.00 | 0.17 ± 0.12 |
| 28 | 0.03 ± 0.03 | 0.00 ± 0.00 | 0.02 ± 0.04 | 0.00 ± 0.00 |
| 56 | 0.04 ± 0.01 | 0.00 ± 0.00 | 0.09 ± 0.09 | 0.05 ± 0.05 |
| 90 | 0.09 ± 0.01 | 0.05 ± 0.08 | 0.04 ± 0.08 | 0.06 ± 0.02 |
| Testosterone (ng/mL) | | | | |
| 0 | 0.00 ± 0.00 | 0.02 ± 0.02 | 0.01 ± 0.01 | 0.02 ± 0.01 |
| 7 | 0.01 ± 0.00 | 0.28 ± 0.17 | 1.02 ± 0.38 | 1.25 ± 0.20 |
| 14 | 0.02 ± 0.01 | 0.34 ± 0.10 | 1.40 ± 0.39 | 1.93 ± 0.39 |
| 28 | 0.01 ± 0.00 | 0.33 ± 0.25 | 1.01 ± 0.15 | 1.54 ± 0.15 |
| 56 | 0.01 ± 0.01 | 0.23 ± 0.11 | 1.21 ± 0.38 | 2.25 ± 0.52 |
| 90 | 0.01 ± 0.00 | 0.50 ± 0.49 | 2.04 ± 0.31 | 2.29 ± 0.46 |
| Estradiol (pg/mL) | | | | |
| 0 | 0.18 ± 0.31 | 0.34 ± 0.59 | 0.46 ± 0.41 | 0.00 ± 0.00 |
| 7 | 0.00 ± 0.00 | 0.00 ± 0.00 | 0.00 ± 0.00 | 0.33 ± 0.58 |
| 14 | 0.00 ± 0.00 | 0.00 ± 0.00 | 0.00 ± 0.00 | 0.34 ± 0.59 |
| 28 | 0.00 ± 0.00 | 0.00 ± 0.00 | 0.46 ± 0.42 | 0.22 ± 0.38 |
| 56 | 0.22 ± 0.38 | 0.20 ± 0.35 | 0.00 ± 0.00 | 0.51 ± 0.46 |
| 90 | 0.31 ± 0.53 | 0.61 ± 0.64 | 1.25 ± 1.51 | 1.08 ± 0.51 |
| Cortisol (ng/mL) | | | | |
| 0 | 15.3 ± 5.73 | 15.4 ± 2.79 | 13.8 ± 1.47 | 18.0 ± 3.56 |
| 7 | 15.8 ± 2.27 | 14.7 ± 3.09 | 15.0 ± 0.72 | 17.7 ± 6.38 |
| 14 | 26.9 ± 19.69 | 15.3 ± 2.71 | 15.4 ± 0.25 | 18.8 ± 7.06 |
| 28 | 17.5 ± 1.58 | 13.0 ± 3.17 | 11.2 ± 1.52 | 9.9 ± 0.65 |
| 56 | 14.5 ± 0.66 | 12.8 ± 2.25 | 11.3 ± 1.88 | 13.6 ± 3.94 |
| 90 | 15.6 ± 3.71 | 13.8 ± 4.30 | 11.0 ± 0.47 | 11.7 ± 0.57 |
| Thyroid Stimulating Hormone (ng/mL) | | | | |
| 0 | 0.16 ± 0.04 | 0.10 ± 0.03 | 0.15 ± 0.02 | 0.19 ± 0.06 |
| 7 | 0.11 ± 0.03 | 0.12 ± 0.01 | 0.10 ± 0.04 | 0.15 ± 0.01 |
| 14 | 0.09 ± 0.03 | 0.14 ± 0.04 | 0.11 ± 0.05 | 0.19 ± 0.05 |
| 28 | 0.15 ± 0.08 | 0.14 ± 0.04 | 0.17 ± 0.07 | 0.14 ± 0.03 |
| 56 | 0.13 ± 0.10 | 0.16 ± 0.10 | 0.10 ± 0.01 | 0.16 ± 0.01 |
| 90 | 0.15 ± 0.02 | 0.11 ± 0.03 | 0.15 ± 0.01 | 0.16 ± 0.05 |
| Thyroid Hormone (T4) (ng/mL) | | | | |
| 0 | 36.2 ± 4.45 | 34.3 ± 0.91 | 35.2 ± 2.25 | 34.9 ± 2.80 |
| 7 | 35.5 ± 4.01 | 34.1 ± 1.50 | 34.6 ± 1.16 | 34.1 ± 3.06 |
| 14 | 34.9 ± 2.80 | 35.0 ± 0.90 | 34.6 ± 1.58 | 34.9 ± 1.56 |
| 28 | 34.9 ± 5.57 | 33.3 ± 0.18 | 34.8 ± 2.42 | 35.1 ± 4.27 |
| 56 | 36.9 ± 5.58 | 34.5 ± 1.47 | 33.1 ± 1.42 | 36.7 ± 3.38 |
| 90 | 35.9 ± 4.47 | 34.9 ± 0.22 | 34.3 ± 2.71 | 35.5 ± 1.70 |
| Luteinizing Hormone (ng/mL) | | | | |
| 0 | 26.9 ± 23.54 | 25.9 ± 14.39 | 41.7 ± 21.99 | 33.1 ± 22.61 |
| 7 | 23.0 ± 17.32 | 22.6 ± 9.67 | 35.8 ± 7.85 | 26.4 ± 32.62 |
| 14 | 31.5 ± 23.09 | 24.9 ± 9.83 | 36.6 ± 18.08 | 54.3 ± 93.30 |
| 28 | 28.2 ± 16.39 | 28.8 ± 10.88 | 52.4 ± 69.12 | 1.2 ± 1.44 |
| 56 | 46.9 ± 36.68 | 29.7 ± 10.78 | 31.9 ± 28.14 | 0.4 ± 0.72 |
| 90 | 42.0 ± 31.85 | 27.5 ± 8.26 | 10.6 ± 10.34 | 0.6 ± 0.56 |

Supplementary Table 4. Comparison of testosterone delivery methods applicable to dogs^1^. Given the limited data in dogs, most information was based on human literature.

| Method^1^ | Form | | Pros | Cons |
| --- | --- | --- | --- | --- |
| Oral  (Testosterone undecanoate^2^) | Capsule | - Ease of administration  - T. undecanoate avoids hepatic metabolism  - Fast reversal | | - Poor availability requiring multiple daily doses in men  - Short half-life  - T. undecanoate requires high-fat meal and has initial high dihydrotestosterone level |
| Transdermal | Gel, lotion | | - Ease of administration  - Fast reversal  - More stable testosterone level | - Daily administration  - Short half-life  - Skin irritation  - Transference to others  - Lack of application sites in dogs  - Possible ingestion in dogs |
| Injection  (Testosterone cypionate^3^) | Oil-based liquid | | - Absorbed by tissue - systemic action  - No transference to others  Weekly subcutaneous injection:  - Less pain  - Ease of administration  - More stable testosterone level | - Possible erythrocytosis  2-6 week intramuscular injection: - Difficult to administer  - Inflammation and pain at injection site  - Fluctuation in testosterone levels |
| Implant | Pellets | | - Long-lasting  - Absorbed by tissue – systemic action  No transference to others | -Requires surgical implantation  -Site infection or pellet extrusion  - Longer half-life - peak then long decay  -Difficult to remove if necessary  -Replacement timing depends on multiple factors (BMI, # pellets)  - Possible erythrocytosis |

^1^ Other methods of testosterone administration, including transdermal patch, buccal, sublingual, nasal, and rectal, are less applicable to dogs.

^2^ New oral testosterone formulations are under development to address the disadvantages of testosterone undecanoate

^3^Other testosterone esters are available, with varying doses, half-lives, and impacts

References: [4-13]

**References**

1. Niswender GD. Influence of the site of conjugation on the specificity of antibodies to progesterone. Steroids. 1973 Sep;22(3):413–24.

2. England BG, Niswender GD, Midgley AR. Radioimmunoassay of estradiol-17β without chromatography. J Clin Endocrinol Metab. 1974 Jan;38(1):42–50.

3. Berndtson WE, Pickett BW, Nett TM. Reproductive physiology of the stallion. IV. Seasonal changes in the testosterone concentration of peripheral plasma. J Reprod Fertil. 1974 Jul;39(1):115–8.

4. Palerme JS, Mazepa A, Hutchins RG, Ziglioli V, Vaden SL. Clinical response and side effects associated with testosterone cypionate for urinary incontinence in male dogs. J Am Anim Hosp Assoc. 2017 Sep 1;53(5):285–90.

5. Brent L, Lissner EA, Kutzler MA. Restoration of reproductive hormone concentrations in a male neutered dog improves health: A case tudy. Top Companion Anim Med. 2021 Nov;45:100565.

6. Ahmad SW, Molfetto G, Montoya D, Camero A. Is oral testosterone the new frontier of testosterone replacement therapy? Cureus [Internet]. 2022 Aug 8 [cited 2024 Jul 22]; Available from: https://www.cureus.com/articles/26237-is-oral-testosterone-the-new-frontier-of-testosterone-replacement-therapy

7. Barbonetti A, D’Andrea S, Francavilla S. Testosterone replacement therapy. Andrology. 2020 Nov;8(6):1551–66.

8. Ohlander SJ, Varghese B, Pastuszak AW. Erythrocytosis following testosterone therapy. Sex Med Rev. 2018 Jan;6(1):77–85.

9. Wang C, Swerdloff RS. Testosterone replacement therapy in hypogonadal men. Endocrinol Metab Clin North Am. 2022 Mar;51(1):77–98.

10. Yin AY, Htun M, Swerdloff RS, Diaz‐Arjonilla M, Dudley RE, Faulkner S, et al. Reexamination of pharmacokinetics of oral testosterone undecanoate in hypogonadal men with a new self‐emulsifying formulation. J Androl. 2012 Mar 4;33(2):190–201.

11. Newell-Price J, Huatan H, Quirke J, Porter J, Daniel E, Mumdzic E, et al. An oral lipidic native testosterone formulation that is absorbed independent of food. Eur J Endocrinol. 2021 Nov 1;185(5):607–15.

12. McFarland J, Craig W, Clarke NJ, Spratt DI. Serum testosterone concentrations remain stable between injections in patients receiving subcutaneous testosterone. J Endocr Soc. 2017 Aug 1;1(8):1095–103.

13. Pastuszak AW, Mittakanti H, Liu JS, Gomez L, Lipshultz LI, Khera M. Pharmacokinetic evaluation and dosing of subcutaneous testosterone pellets. J Androl. 2012 Sep 10;33(5):927–37.
